# Short‐term starvation stress at young adult stages enhances meiotic activity of germ cells to maintain spermatogenesis in aged male *Caenorhabditis elegans*

**DOI:** 10.1111/acel.12930

**Published:** 2019-02-28

**Authors:** Wan‐Yi Chou, Yu‐Chun Lin, Ying‐Hue Lee

**Affiliations:** ^1^ Laboratory of Molecular Pathology, Institute of Molecular Biology Academia Sinica Taipei Taiwan

**Keywords:** anaphase‐promoting complex/cyclosome (APC/C), CDC‐20, meiosis, short‐term starvation, spermatogenesis, stress response hormesis

## Abstract

To survive and reproduce, living organisms must evolve numerous mechanisms to re‐adjust their physiology when encountering adverse conditions that subject them to severe stress. We found that short‐term starvation (STS) stress in young adult male *Caenorhabditis elegans* can significantly improve their vitality (relative to nonstressed males) when they are aged. In addition, we found that stress‐treated aged males maintained reproductive activity equivalent to young males, whereas nonstressed aged males quickly lost reproductive ability. STS stress can preserve sperm number and quality in aged male worms. Spermatogenesis involves germ cell mitosis and meiosis. We found that germ cell meiotic activity is more sensitive to aging than mitotic activity and is declining rapidly with age. We examined the role of numerous factors important for spermatogenesis on STS‐preserved spermatogenesis during aging. Our results show that mutant strains deficient in anaphase‐promoting complex/cyclosome (APC/C) function fail to exhibit the STS stress‐enhanced spermatogenesis found in wild‐type *N2* worms, suggesting that the mechanism underlying starvation‐induced spermatogenesis involves the APC/C complex, a conserved ubiquitin‐protein ligase E3 complex. Furthermore, transgenic expression of FZY‐1/CDC‐20, a coactivator of APC/C, ameliorated the age‐associated decline of meiosis, similar to the hormetic effect of STS.

## 1 INTRODUCTION

For species maintenance, living organisms must survive and reproduce. In nature, lack of food is one of the major and most frequent threats to survival. In response to the stress of food deprivation, living organisms have evolved numerous mechanisms to re‐adjust their physiological and metabolic activities to survive the stress period (McCue, 2010; Wang, Hung, & Randall, 2006). For example, at the organismic level, the soil nematode *Caenorhabditis elegans* undergoes developmental arrest during larval stages, which allows them to preserve energy and endure long periods of starvation and stress (Baugh, 2013; Riddle, 1997), and at the cellular level, structural proteins are targeted for degradation via lysosome and autophagy pathways for use as an alternative energy source (Gelino et al., 2016; Singh & Cuervo, 2011).

Since multiple adverse conditions occur in nature, animals that survive one stress must recover quickly before they experience another. This raises the possibility that to ensure quick recovery at the poststress ages, organisms must enhance their strength while re‐adjusting their physiological and biochemical activities to cope with a current stress. Consequently, survivors of stress conditions might become stronger than their unstressed counterparts, a phenomenon named “stress response hormesis.” Hormesis describes the beneficial effects of sublethal stress on organisms, which can enhance subsequent stress resistance and even increase life expectancy (Cypser & Johnson, 2002; Cypser, Tedesco, & Johnson, 2006; Gems & Partridge, 2008). Hormesis in the form of food or calorie restriction (CR) has been found in divergent species (Weindruch, 1996), effectively extending lifespan and delaying onset of age‐related disorders without genetic alteration (L'opez‐LIuch & Navas, 2016; Nakagawa, Lagisz, Hector, & Spencer, 2012; Salvatore et al., 2016).

One of the most crucial physiological functions of an organism is its capacity to reproduce. Thus, for a species to thrive, stress‐induced hermetic effects should endow numerous physiological and biochemical benefits on an organism to bolster its reproductive capacity. However, despite there being well‐reported hormetic effects on aging and lifespan, the impacts of CR on reproduction have been reported to be minimal and are somewhat contradictory (Brito et al., 2007; Moatt, Nakagawa, Lagisz, & Walling, 2016; Selesniemi, Lee, & Tilly, 2008; Sitzmann et al., 2014).

Here, we demonstrate that short‐term starvation (STS) stress in young adult male *C. elegans* effectively prevents age‐related declines in sperm production, and repetitive fasting can further enhance this effect. In addition, the underlying molecular mechanism involves STS stress‐enhanced meiotic activity during spermatogenesis, perhaps mediated by FYZ‐1/CDC‐20, a coactivator of anaphase‐promoting complex/cyclosome (APC/C) that plays a key role in regulating meiosis.

## 2 RESULTS

### 2.1 STS stress treatment of early adult male *C. elegans* enhances vitality and reduces mortality during aging

**Figure 1:**
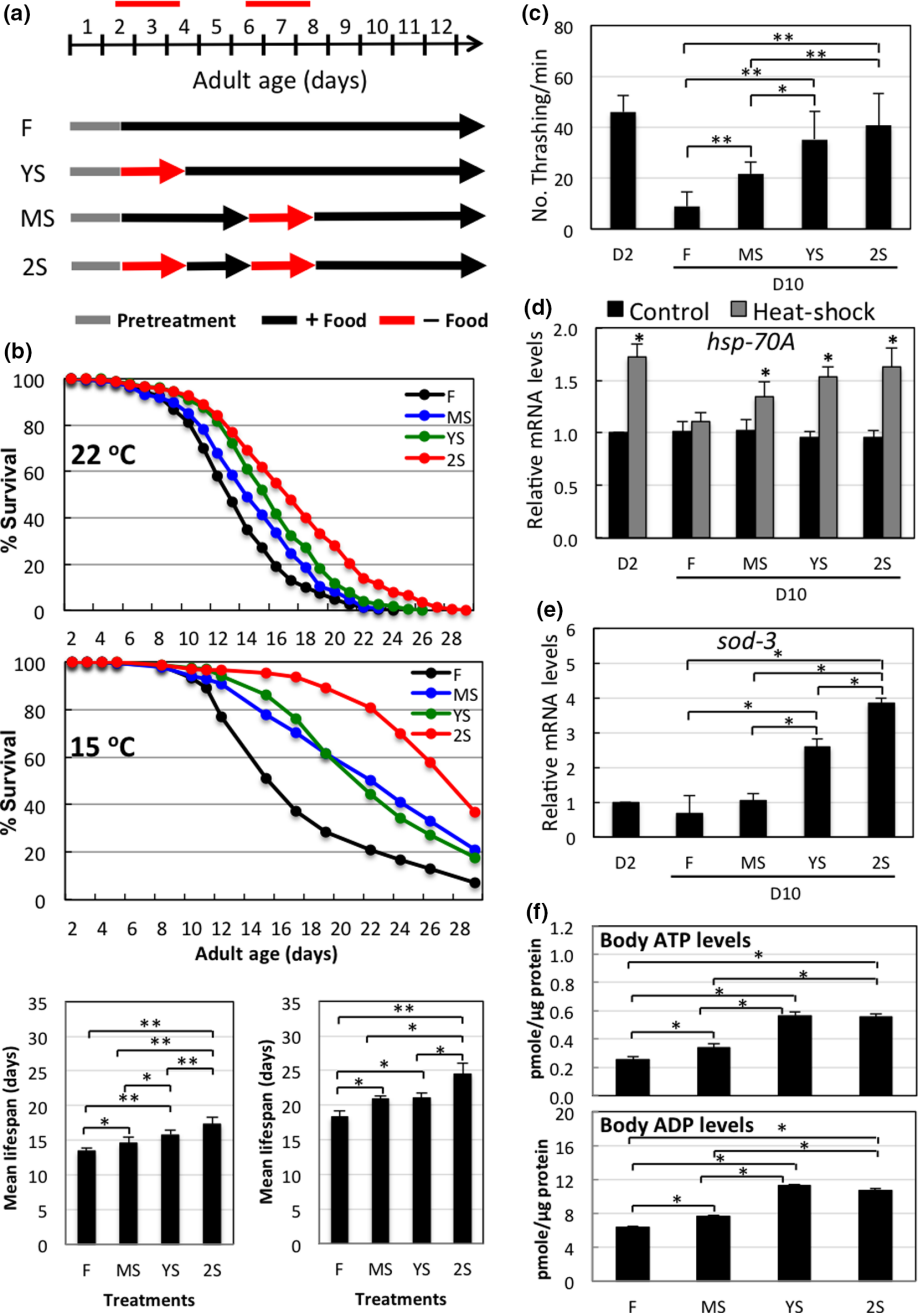
Short‐term starvation (STS) stress preserves viability and vitality in adult male *C. elegans* during aging. (a) Schematic of STS treatments in adult male *C. elegans* maintained at 22°C. The first day of adulthood is denoted D1. Y: young‐age; M: mid‐age; S: starved. (b) Survival curves of *N2* adult male worms maintained at either 15 or 22°C and that received various STS treatments as shown in Figure 1a. Data represent mean of *n* = 4 (15°C, middle panel) and *n* = 5 (22°C, upper panel), respectively. The survival curves for all the replicates are shown in Supporting Information Figure S1. For mean lifespan (bottom panel), data represent mean ± *SD*. Difference between two indicated groups: *, *p *< 0.05, **, *p *< 0.01*.* (c) Physical activity of STS stress‐treated adult male worms. D10 adult male worms subjected to various STS stress treatments as shown in Figure 1a were assayed for their thrashing activity. Data represent mean ± *SD*, *n* ≥ 10. Difference between two indicated groups: *, *p *≤ 0.05; **, *p *≤ 0.001*.* (d) qRT‐PCR analysis of *hsp‐70A* mRNA levels in D10 adult male worms subjected to various STS stress treatments as shown in Figure 1a and treated at 30°C for 3 hr to induce *hsp‐70A*expression. Data represent mean ± *SD*, *n* = 4. *Different from fed control (F) value of same group, *p *≤ 0.001*.* (e) qRT‐PCR analysis of *sod‐3* mRNA levels in D10 adult male worms subjected to various STS stress treatments as shown in Figure 1a. Data represent mean ± *SD*, *n* = 4. Difference between two indicated groups: *, *p *≤ 0.001*.* (f) Body ATP levels of D10 adult male worms subjected to various STS stress treatments as shown in Figure 1a. Data represent mean ± *SD*, *n* = 3. Difference between two indicated groups: *, *p *≤ 0.001

Previously, we showed that adult male *C. elegans* exhibit different phases of metabolic readjustment in response to different durations of food deprivation (Tan, Luo, Ho, & Lee, 2011). Here, we examined the hormetic effect of STS stress on the physiology of adult male *C. elegans* at poststress ages. We starved male worms at various adult stages for 48 hr (as illustrated in Figure 1a) and then monitored their survival rate. We found that STS stress for 48 hr, either at an early (YS) or mid‐stage (MS), reduced the mortality rate of male worms (maintained at 22°C) in the poststress period compared to control males and that repetitive fasting (2S) was even more efficient at reducing mortality rate (Figure 1b; Supporting Information Figure S1), suggesting a cumulative effect. This reduction in mortality was further enhanced for male worms kept at 15°C (Figure 1b; Supporting Information Figure S1). Insignificant longevity responses to CR and intermittent fasting have been previously reported (Honjoh, Ihara, Kajiwara, Yamamoto, & Nishida, 2017), but we found that STS stress efficiently increases lifespans of adult male worms (Figure 1b). This discrepancy is likely due to differences in the culturing systems (solid agar plate vs. liquid) and the starvation treatment (lifelong vs. transient) used.

At day 10 (D10), we analyzed worms subjected to prior stress for their viability and responses to other subsequent stresses. First, to assess the locomotor activity of stressed worms, we measured the thrashing frequency of worms in a liquid system. At D10, thrashing frequency of control worms was reduced to <20% that of worms at D2. However, subjecting worms to early stress (YS) rescued the decline in movement observed for fed control worms (F), and repetitive stress again was the most effective means of preventing this aging‐associated decline (Figure 1c). To assess the ability of STS‐treated worms to respond to other stresses, we heat‐shocked previously starved D10 male worms and analyzed their expression levels of the heat‐shock gene *hsp‐70A*. As expected, heat‐shock significantly elevated expression levels of *hsp‐70A* mRNA in young adult D2 worms, but failed to effectively stimulate *hsp‐70A* expression in D10 worms. Early STS stress treatment (YS) and particularly the repetitive STS treatment (2S) enhanced the stimulatory effect of heat‐shock on *hsp‐70A* expression (Figure 1d). Expression levels of *sod‐3*, which encodes an anti‐oxidant superoxide dismutase and represents ability to eliminate oxidative stress, were higher in YS‐ and 2S‐treated worms than in control males (Figure 1e). Energy decline is associated with aging. Indeed, ATP levels decline with age in adult male worms (Supporting Information Figure S2A). Nevertheless, early STS treatment significantly enhanced ATP levels in aged males (Figure 1f). These results demonstrate that STS stress treatment at the early adult stages of male worms has beneficial and lasting effects on their viability.

### 2.2 STS extends spermatogenesis and reproductive span in aging male *C. elegans*

**Figure 2:**
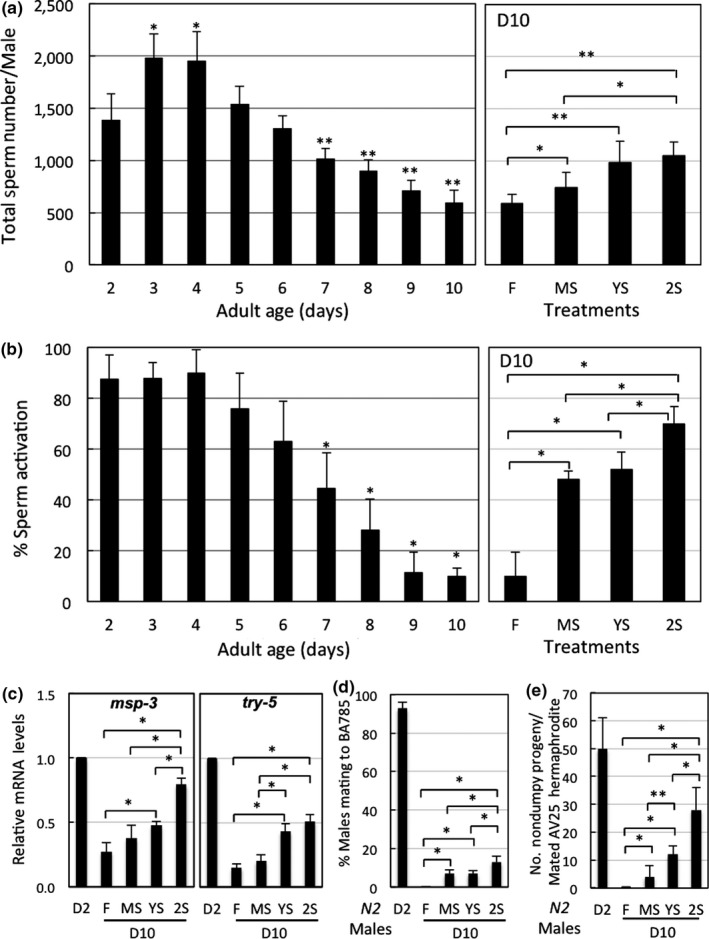
Short‐term starvation (STS) stress‐preserved vitality is coupled to reproductive fitness in adult male *C. elegans* during aging. (a) Total sperm numbers in *N2* adult male worms of different ages maintained at 22°C, and of different STS stress‐treated worms as shown in Figure 1a. Data represent mean ± *SD*, *n* ≥ 10. Left panel, *different from day 2 level, *p *≤ 0.05; **different from day 2 level, *p *≤ 0.001. Right panel, difference between two indicated groups: *, *p *≤ 0.05; **, *p *≤ 0.001*.* (b) Sperm quality assayed in an in vitro sperm activation system using triethanolamine (TEA). Data represent mean ± *SD*, *n* ≥ 10. Left panel, *different from day 2 level, *p *≤ 0.001. Right panel, difference between two indicated groups: *, *p *≤ 0.001*.* (c) Relative mRNA levels of *msp‐3* and *try‐5* by qRT‐PCR analysis*.*Data represent mean ± *SD*, *n* = 4. Difference between two indicated groups: *, *p *≤ 0.001*.* (d) Copulatory activity in D10 adult male worms. Data represent mean ± *SD*, *n* = 4. Difference between the two indicated groups: *, *p *≤ 0.001*.* (e) Fertilization ability in D10 adult male worms. Data represent mean ± *SD*, *n* ≥ 30. Difference between the two indicated groups: *, *p *≤ 0.05. **, *p *≤ 0.001

Total sperm number in male worms declines rapidly from D4 (Figure 2a). Similarly, sperm quality diminishes with age, as determined by their decreasing ability to form pseudopods upon triethanolamine treatment in vitro and their decreased mRNA levels of *msp‐3* and *try‐5* that encode the major sperm protein for pseudopod formation and a semen protease required for sperm activation (Figure 2b,c; Supporting Information Figure S2B). However, STS at early adult stages prevents this age‐associated decline of sperm quality and quantity (Figure 2a,b). To ensure D10 males still actively copulate and sire, we allowed D10 males to interact with hermaphroditic *spe‐8* mutants of the BA785 [*spe‐8(hc40)*] and AV125 [*spe‐8(hc40)*; *dpy‐4(e1166)*] strains, which lack nonreceptor tyrosine kinase SPE‐8 function and cannot transactivate their own sperm for self‐fertilization. However, upon mating with male worms, sperm from hermaphrodites of these strains can be activated by seminal fluid from wild‐type *N2* males, allowing fertilization (L'Hernault, Shakes, & Ward, 1988; Muhlrad, Clark, Nasri, Sullivan, & LaMunyon, 2014). AV125 worms also carry a mutation in their *dpy‐4* gene and produce dumpy progeny if self‐sperm is activated and used. We found that more than 90% of the young *N2* males (D2) we tested successfully copulated with *spe‐8* mutant hermaphrodites, whereas D10 *N2* males did not copulate with these mutants. However, prior STS stress improved the copulatory activity of D10 males (Figure 2d). In addition, sperm from STS‐treated D10 males could fertilize mutant hermaphrodites to produce offspring (Figure 2e). Repetitive stress (2S) treatment was again the most effective in terms of prolonging reproductive ability.

### 2.3 Meiotic activity during spermatogenesis declines rapidly in young adult male *C. elegans*

**Figure 3:**
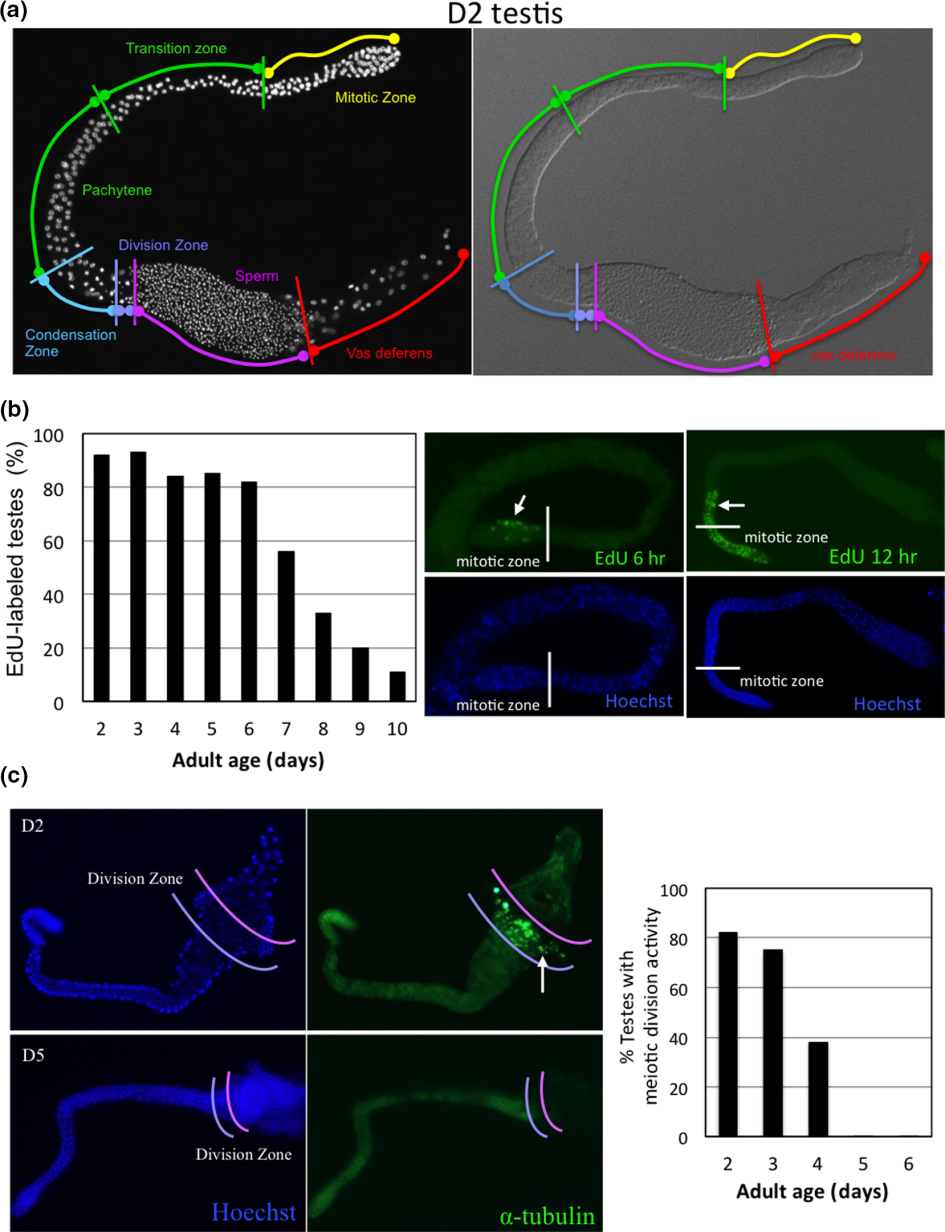
Meiotic, not mitotic, activity of spermatogenesis declines rapidly in young adult male *C. elegans*. (a) Anatomy of the *C. elegans* male testis and germ cell nuclei morphology. Right panel, DIC image of an intact testis isolated from a D2 *N2*adult male worm. Left panel, nuclei visualized by Hoechst 33342 staining. Regions of the testis are indicated. (b) Left panel, EdU incorporation rate in testes of *N2* adult male worms of different ages maintained at 22°C. More than 50 testes from each age‐group were examined for the presence of EdU signal after labeling for 24 hr. Right panel, fluorescent images of EdU‐labeled and Hoechst 333‐stained testes. Arrow indicates EdU‐labeled nuclei. (c) Right panel, meiotic division activity in the testes of *N2* adult male worms of different ages maintained at 22°C. More than 50 testes from each age‐group were analyzed. Left panel, immunofluorescent staining of meiotic α‐tubulin in testes of D2 and D5 *N2* adult males. Arrow indicates an α‐tubulin spindle

Gametogenesis involves mitosis and meiosis of germ cells. To examine mitotic activity during spermatogenesis over the course of aging in male *C. elegans*, we analyzed the ability of their testes to incorporate EdU (5‐ethynyl‐2′‐deoxyuridine) into DNA to assess DNA synthesis activity in various age cohorts. The gonads of male worms can be divided into several zones, each containing nuclei with distinct morphologies, as illustrated in Figure 3a. We found that EdU incorporation first appears in nuclei of the mitotic zone, before later extending into nuclei of other zones (Figure 3b, right panel). We collected the testes from male worms of the various age cohorts pretreated in EdU solution for 24 hr and assessed EdU signal in nuclei of the mitotic zone. The proportion of testes still possessing mitotic activity declined rapidly from D6 and, at D10, only 10% of examined testes displayed EdU signal (Figure 3b, left panel).

To assess spermatogenic meiotic activity during aging, we analyzed meiosis in the division zone of testes by immunocytochemistry using an antibody against α‐tubulin—a component of the division spindle for both meiosis I and II—to visualize division spindles. At D2, division spindles were readily detected in more than 80% of the testes we examined but, from D3, the number of testes still exhibiting meiotic division rapidly diminished. At D5, division spindles could no longer be detected in any of the testes we examined (Figure 3c). We obtained similar results when we monitored meiosis in the testes of live transgenic *AZ244* worms that carry a transgene expressing an α‐tubulin‐GFP fusion protein (Supporting Information Figure S3). These results indicate that meiosis during spermatogenesis is affected earlier during aging than mitotic activity.

### 2.4 STS preserves meiotic activity of spermatogenesis in aging male *C. elegans*

**Figure 4:**
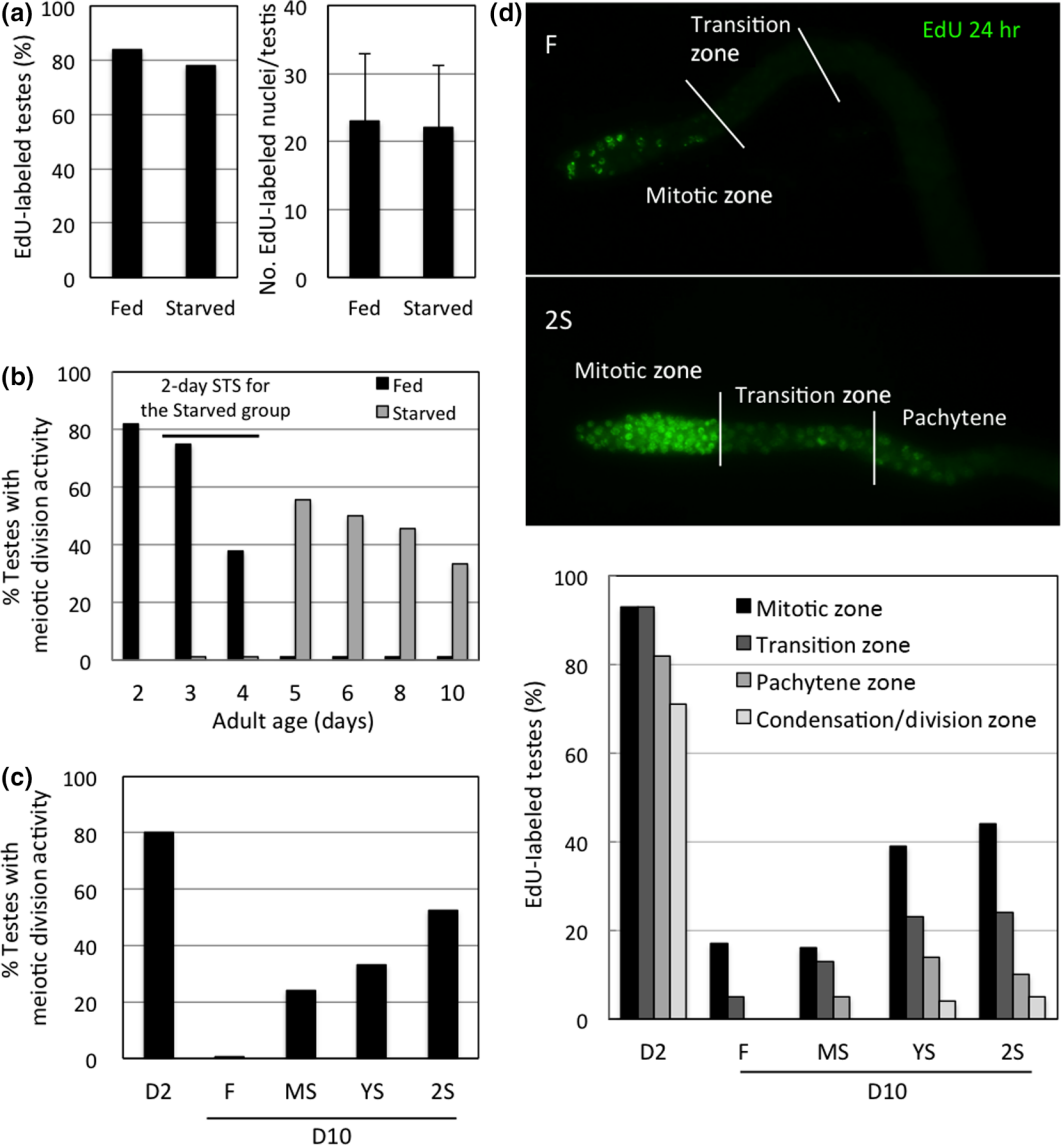
Short‐term starvation (STS) abolishes meiotic activity during stress, but markedly enhances this activity during poststress ages in adult male *C. elegans*. (a) EdU incorporation rate in testes of D4 adult males that had been starved for 2 days at 22°C. More than 50 testes from each age‐group were examined for the presence of EdU‐labeled nuclei after labeling for 6 hr. (b) Meiotic activity in testes of adult males of different ages maintained at 22°C. In the starved group, adult males were starved from D2 to D4. More than 50 testes from each age‐group were analyzed. (c) Meiotic division activity in testes of D10 adult males subjected to STS treatments earlier as shown in Figure 1a and maintained at 22°C. More than 50 testes from each age‐group were analyzed. (d) Translocation efficiency of EdU‐labeled nuclei in testes of D10 adult males previously subjected to different STS stress treatments as shown in Figure 1a. More than 50 testes from each age‐group were examined for the localization of EdU‐labeled nuclei after labeling for 24 hr

To examine how STS stress preserves spermatogenesis in aged male worms, we first analyzed their mitotic and meiotic activities upon STS stress. Interestingly, based on both the percentage of testes exhibiting EdU incorporation and the number of EdU‐positive nuclei in each testis, we found that the ability of testis to incorporate EdU into nuclei of the mitotic zone was similar between STS‐stressed and control males (Figure 4a; Supporting Information Figure S4A). In contrast, 2 days of STS effectively abolished the presence of α‐tubulin spindles in the division zones of testes (Figure 4b), suggesting that starvation primarily affects meiotic activity during spermatogenesis, perhaps to reduce sperm production during the stress period. Indeed, we found that total sperm counts were significantly reduced in males starved for 2 days (Supporting Information Figure S4B). Furthermore, total numbers of nuclei from the mitotic to pachytene zones of testes from starved males were increased, particularly in the pachytene zone. In contrast, total nuclei in the condensation/division zones were significantly reduced (Supporting Information Figure S4B). The increased numbers of nuclei before the meiotic division zone likely reflect an accumulation of germ cell nuclei due to unaffected mitotic activity and inhibition of their meiosis. Similarly, the significantly fewer nuclei in the condensation/division zones are likely due to inhibition of nuclei in pachytene from entering the condensation/division zones for meiotic division.

Despite meiotic division of spermatogenesis being completely inhibited during STS stress, once released from that stress, the testes from the stressed males recovered meiotic division. Furthermore, the rapid decline with age of meiotic activity seen in the control testes was not apparent in the testes of stressed males (Figure 4b), indicating that STS stress elicits a preservation effect that prevents rapid decline in meiotic activity. This preservation effect was observed in all STS‐treated males but, as expected, the repetitive 2S treatment was the most effective (Figure 4c).

We then examined whether STS stress could also preserve the mitotic activity of spermatogenesis in the poststress period. Indeed, we found that the number of testes that incorporated EdU into nuclei of their mitotic zone at D10 was increased in YS‐ or 2S‐treated males (Figure 4d). Notably, the number of EdU‐labeled nuclei that had been translocated to proximal regions of testes was markedly increased in the testes of stressed males (especially 2S males), indicating that germ cells still entered meiosis for sperm formation. In addition, STS stress facilitated translocation of EdU‐labeled nuclei from the mitotic to division zones within 24 hr, since EdU‐labeled nuclei in the control testes largely remained in the mitotic zone, suggesting that STS stress is also effective in facilitating germ cells to enter into the meiotic cycle. Thus, our findings indicate that STS stress prevents both mitotic and meiotic activities from rapidly declining with age, maintaining sperm production in poststress ages.

### 2.5 APC/C complex activity is required for STS stress‐preserved spermatogenesis in aged male *C. elegans*

To understand the molecular mechanism by which STS stress exerts its hormetic effect to preserve spermatogenic activity during aging, we first used mutant worms to examine whether various factors (listed in Table 1) are functionally required for STS stress‐preserved sperm production in aged males. Based on our observations that meiotic activity was better preserved by STS stress, we focused on factors involved in meiotic regulation. Temperature‐sensitive (ts) mutants were kept at 15°C, whereas non‐temperature‐sensitive mutants were kept at 22°C. Non‐temperature‐sensitive mutant males were collected on D1 and fasted on D2, as shown in Figure 1a. For ts mutant worms, adult males were collected on D1, maintained at 25°C on D2 for 1 day, before being subjected to fasting on D3 and following the same scheme as indicated in Figure 1a, but were maintained at 22°C thereafter. Our criterion for considering that a gene is involved in STS stress‐preserved spermatogenesis of aged males was that STS stress could not elevate sperm production in the respective mutant males, as in *N2* males (Figure 2a, right panel). Despite assessing many factors previously reported to play roles in spermatogenesis, worms mutated for the majority of these factors had sperm counts similar to those of *N2* control males (Table 1), suggesting that these factors might exhibit functional redundancy with other factors, while mutant lines possessing defects in *ceh‐18*, *emb‐30,* or *spe‐26* had significantly lower sperm counts than *N2* controls, indicating that these factors might play a crucial role in *C. elegans* spermatogenesis.

**Table 1:** Total sperm counts in D10/D11 mutant male *C. elegans* that were previously subjected to an STS stress

Strains and *genes*	Total sperm count (mean ± *SD*, *n* = 8–12)					Required for STS stress‐preserved spermatogenesis
	D2	D10 (or D11 for ts strains)				
		F	MS	YS	2S	
Wild‐type						
*N2*(22°C)	1,319 ± 280	482 ± 306	746 ± 237	811 ± 381	990 ± 349^a^,*	Control
*N2*(treated as for ts mutants)	1,835 ± 436^b^	999 ± 359	1,260 ± 250	1,962 ± 563***	2,247 ± 677***	Control
Promotion of meiotic entry^c^						
BS14 *gld‐1(q266)/nDf24*	1,967 ± 459	325 ± 275	569 ± 251	787 ± 388*	1,549 ± 373***	No
RB1181 *gld‐2(ok1117)*	1,520 ± 395	292 ± 234	421 ± 218	562 ± 201*	820 ± 398**	No
WM100 *cks‐1(ne549)*(ts)	1,887 ± 332	796 ± 225	887 ± 302	937 ± 166	1,317 ± 492**	No
Regulation of meiotic progression						
WM99 *cdk‐1(ne2257)*(ts)	1,745 ± 208	2,351 ± 621	2,689 ± 367	2,603 ± 694	2,469 ± 403	Yes
EU1441 *plk‐1(or683)*(ts)	2,111 ± 383	2,775 ± 702	2,546 ± 374	2,557 ± 347	2,263 ± 349*	Yes
Chromosome homolog pairing						
CA324 *zim‐1(tm1813)*	1,345 ± 348	1,232 ± 417	1,081 ± 310	968 ± 447	998 ± 436	Yes
Meiotic recombination (chromosome stability and segregation fidelity)						
CE1255 *cep‐1(ep347)*	1,133 ± 299	748 ± 340	861 ± 282	1,097 ± 344**	1,182 ± 305**	No
Meiotic anaphase initiation—APC/C complex for ubiquitin‐mediated proteolysis						
*HY604* *mat‐1(ye121)*(ts)	1,915 ± 452	2,551 ± 650	2,571 ± 624	2,455 ± 565	2,621 ± 590	Yes
*DS98* *mat‐2(ax102)*(ts)	859 ± 211	1,150 ± 268	805 ± 219	1,068 ± 340	785 ± 165	Yes
HY601 *mat‐3(or344))*(ts)	844 ± 169	484 ± 263	398 ± 208	288 ± 160*	190 ± 114***	Yes
MJ57 *emb‐1(hc57)*(ts)	882 ± 221	189 ± 152	210 ± 132	206 ± 181	172 ± 157	Yes
HY621 *emb‐27(ye143)*(ts)	1,233 ± 259	1,785 ± 410	1,994 ± 531	1,981 ± 615	1,815 ± 474	Yes
DG627 *emb‐30(tn377)*(ts)	314 ± 87	427 ± 100	354 ± 100	435 ± 132	423 ± 123	Yes
RB622 *fzr‐1(ok380)*	1,420 ± 330	682 ± 216	698 ± 341	713 ± 223	749 ± 318	Yes
VC147 *apc‐10 & tag‐31(gk143)*	410 ± 230	138 ± 132	218 ± 220	270 ± 297	423 ± 246*	No
Meiotic anaphase initiation—non‐APC/C complex for ubiquitin‐mediated proteolysis						
DW102 *brc‐1(tm1145)*	2,091 ± 296	1,114 ± 480	737 ± 271*	714 ± 337*	863 ± 265	Yes
EU640 *cul‐2(or209)*(ts)	1,557 ± 576	1,192 ± 465	999 ± 331	1,808 ± 579**	1,941 ± 689**	No
Meiotic spindle formation						
EU2697 *mei‐1(or1178)*(ts)	1,621 ± 404	1,919 ± 510	1,821 ± 394	1,907 ± 326	2,540 ± 416**	No
Meiotic division						
RB647 *cdc‐25.3(ok358)*	1,952 ± 490	1,867 ± 646	1,519 ± 422	1,616 ± 599	1,614 ± 411	Yes
Meiotic maturation (of oocytes)						
RB1103 *ceh‐18(ok1082)*	779 ± 193	254 ± 100	361 ± 156	324 ± 156	434 ± 212**	No
Meiotic maturation (spermatocyte cytoskeletal protein)						
BA821 *spe‐26(hc138)*(ts)	549 ± 200	490 ± 158	341 ± 220	243 ± 173***	196 ± 116***	Yes

Notes

D2: day 2; F: fed control; MS: starvation at mid‐stage; ts: temperature‐sensitive strains; YS: starvation at early stage; 2S: twice starvation (see Methods and Materials).

a Total sperm counts are presented as mean ± *SD*, *n* = 10–15.

b Culture at low temperature (15°C) increases sperm number in male worms.

c References for each gene used in this study are listed in Supporting Information Table S1.

* *p *< 0.05,

** *p *< 0.01,

*** *p *< 0.001, two‐tailed Student's *t test*, compared to the F group of D10 or D11 worms of the same strain.

In wild‐type *N2* males, total sperm number declines with age from D4 (Figure 2a). However, mutant lines carrying defects in *cdk‐1*, *plk‐1*, *mat‐1*, *mat‐2*, *emb‐27*, *emb‐30*, and* mei‐1* did not exhibit this age‐associated decline nor did they respond to STS treatments to increase their sperm production. Based on our criterion aforementioned for evaluating whether a gene is involved in STS stress‐preserved spermatogenesis, these genes are considered to be required, despite that their respective mutant worms behave differently from wild‐type *N2* worms in age‐related reduction in sperm numbers.

We assessed eight of the 15 components of the APC/C complex, a ubiquitin‐protein ligase E3 complex (Yeong, 2004; Alfieri et al., 2016; Zhang et al., 2016; Supporting Information Table S2). APC/C is conserved across species, and it initiates the separation of sister and homologous chromatids by proteasomal degradation of connecting factors such as securin during both mitosis and meiosis (Acquaviva & Pines, 2006; Sprayko et al., 2000). We found that mutant worms with a deficiency of EMB‐30 or APC‐10 exhibited severely reduced sperm counts (<20% that of young adult males in *N2* worms), and defects in MAT‐2, MAT‐3, or EMB‐1 had an even greater effect (approx. 50% reduction in sperm number compared to levels in *N2* control worms). The remaining three APC/C complex factors we assessed did not appear to affect sperm production. Of the eight APC/C components we tested, only APC‐10 was not required for the hormetic effect of STS stress on sperm production. We then examined the remaining APC/C components (listed in Table 2) using an RNA interference (RNAi) approach, since loss‐of‐function mutants for these genes are neither viable nor fertile. When separately knocked down, neither GFI‐3 nor SUCH‐1 reduced the starvation‐induced hormetic effect on sperm production. However, these two proteins are APC‐5 paralogs and are functionally redundant in meiotic division (Stein, Nesmith, Ross, & Golden, 2010). Accordingly, GFI‐3 and SUCH‐1 might also be functionally redundant for the mechanism responsible for the STS‐induced hormetic response in sperm production. Indeed, when we simultaneously knocked down both GFI‐3 and SUCH‐1, we observed a reduction in STS stress‐preserved sperm production compared to that seen for individual RNAi targeting. APC‐2, APC‐11, and APC‐10 form the catalytic core of the APC/C complex, allowing it to catalyze ubiquitination of its substrates (Alfieri et al., 2016; Tang et al., 2001). Surprisingly, based on results from individual knockdown or mutation, none of these subunits appear to play a role in STS stress‐induced sperm production, raising the possibility that these three subunits are also functionally redundant for STS‐induced hormesis. Indeed, when all three catalytic core subunits were simultaneously knocked down by RNAi, sperm production under STS stress was reduced to insignificant levels (Table 2), confirming that these subunits are functionally redundant.

**Table 2:** Total sperm counts in RNAi‐treated male *C. elegans* at day 15 that were previously subjected to an STS stress

RNAi‐treated males (at 15°C)	Total sperm counts (mean ± *SD*, *n* = 8–12)					Required for STS stress‐preserved spermatogenesis
	D4	D15				
		F	MS	YS	2S	
Nontargeting controls						
OP50 bacteria	2,405 ± 413	1,648 ± 395	1,545 ± 315	2,225 ± 497^a^^,^ **	2,508 ± 458***	
L4440 vector	1,900 ± 236	1,661 ± 221	1,959 ± 311*	2,479 ± 307***	2,423 ± 484**	
Targeting controls						
*mat‐3*	2,886 ± 608	2,398 ± 314	1,914 ± 251**	1,705 ± 354**	1,443 ± 492***	Yes
*fzr‐1*	2,406 ± 406	3,281 ± 455	2,944 ± 378	2,564 ± 381*	2,470 ± 392**	Yes
*apc‐10*	2,660 ± 550	1,579 ± 563	1,799 ± 514	2,002 ± 466*	2,659 ± 475***	No
Individually targeted genes						
*apc‐2*	2,693 ± 470	1,777 ± 493	1,566 ± 639	2,025 ± 318	2,282 ± 460*	No
*apc‐11*	2,559 ± 481	1,219 ± 238	1,361 ± 291	1,717 ± 470*	1,799 ± 428**	No
*gfi‐3*	2,013 ± 432	1,448 ± 360	1,406 ± 218	1,820 ± 290*	1,986 ± 487*	No
*Such‐1*	2,211 ± 432	1,383 ± 295	1,260 ± 259	1,690 ± 309*	2,066 ± 365*	No
*mat‐4*	2,524 ± 441	2,711 ± 574	2,542 ± 334	2,409 ± 413	2,492 ± 306	Yes
*apc‐17*	2,312 ± 386	2,544 ± 227	2,472 ± 169	2,394 ± 142	2,421 ± 271	Yes
*fzy‐1*	2,300 ± 280	2,272 ± 312	2,198 ± 357	2,333 ± 311	2,325 ± 226	Yes
Multi‐targeted genes						
*apc‐2*; *apc‐10*; *apc‐11*	2,135 ± 384	1,886 ± 430	2,061 ± 448	2,098 ± 492	2,117 ± 322	Yes
*gfi‐3;such‐1*	2,358 ± 252	2,008 ± 367	2,054 ± 298	2,320 ± 329	2,237 ± 264	Yes

Notes

D4: day 4; F: fed control; MS: starvation at mid‐stage; YS: starvation at early stage; 2S: twice starvation.

a Total sperm counts are presented as mean ± *SD*, *n* = 10–15.

* *p *< 0.05,

** *p *< 0.01,

*** *p *< 0.001, two‐tailed Student's *t test*, compared to the F group of D15 worms of the same strain.

### 2.6 STS stress preserves the expression levels of APC/C subunits during aging

**Figure 5:**
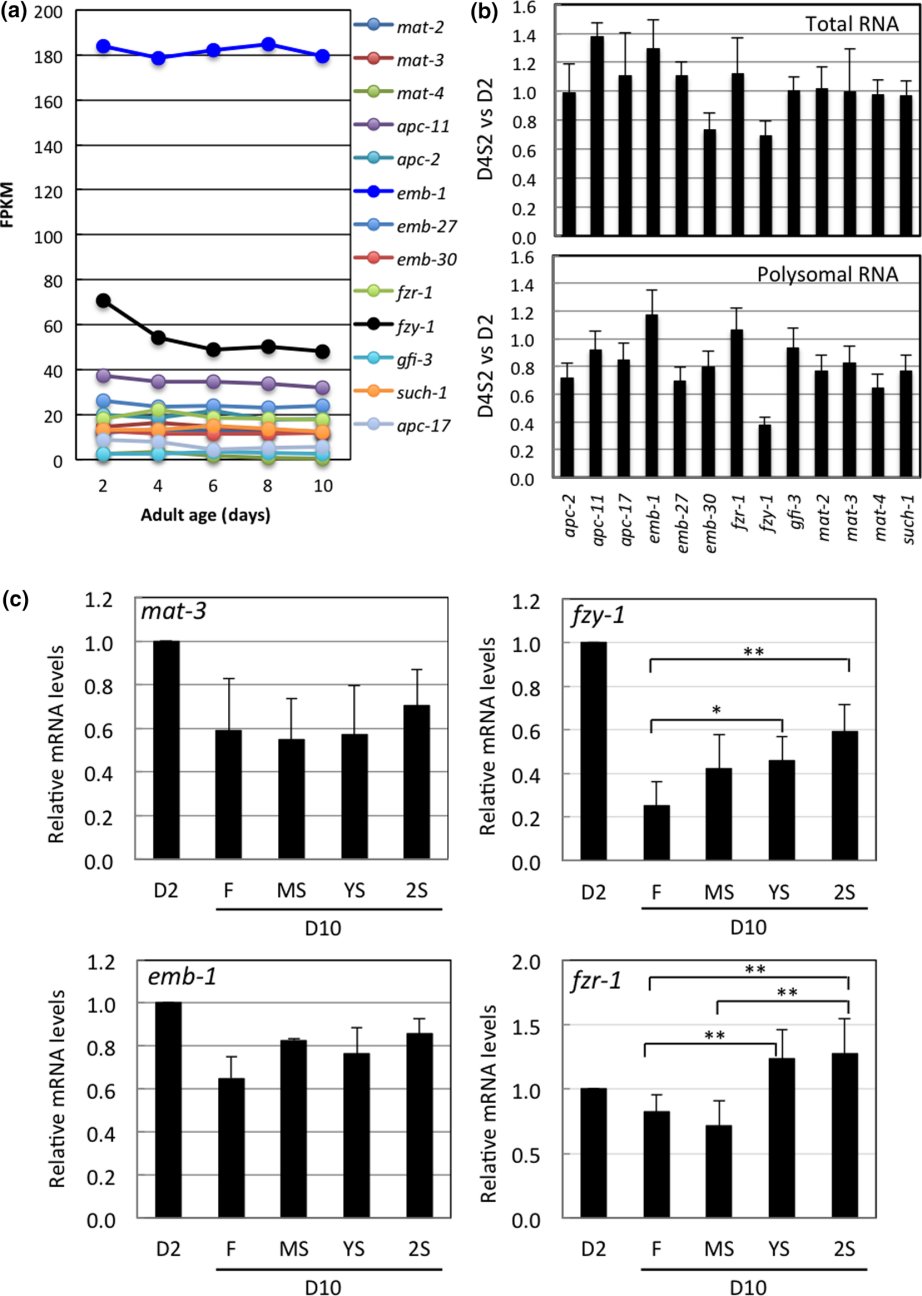
Transcriptional expression of *fzy‐1/cdc‐20* is a target of aging and STS stress. (a) RNA‐seq analysis of expression levels of APC/C subunits in D4 *N2* adult male worms starved for 2 days. Data represent mean ± *SD*, *n* = 3. (b) RNA‐seq analysis of expression levels of APC/C subunits in D4 *N2* adult male worms starved for 2 days (D4S2). The ratio of mRNA levels from starved worms (D4S2) over levels before STS stress treatment (D4) was calculated. Data represent mean ± *SD*, *n* = 3. (c) qRT‐PCR analysis of mRNA levels of APC/C subunits in testes isolated from D10 adult males previously subjected to an STS stress treatment as shown in Figure 1a. qRT‐PCR analysis of the indicated genes was normalized against *sgo‐1* expression levels. Data represent mean ± *SD*, *n* = 4. Difference between two indicated groups: *, *p *≤ 0.05*.* **, *p *≤ 0.01

We then examined whether APC/C subunits are transcriptionally regulated during stress and aging. We first used RNA‐seq analysis to evaluate transcript levels in adult male worms of various ages. As shown in Figure 5a, the transcripts of all subunit genes apart from *fzy‐1* were maintained at similar levels at all stages examined. Transcript levels of *fzy‐1* were highest on day 2, but declined rapidly on days 4–6, after which they remained stable, suggesting that *fzy‐1* transcript levels are impacted by aging.

FZY‐1 is the *C. elegans* ortholog of mammalian CDC‐20, and it is one of two APC/C coactivators. APC/C remains inactive until it associates with either FZY‐1/CDC‐20 or FZR‐1/CDH‐1 (Alfieri, Zhang, & Barford, 2017). The declining transcript levels of *fzy‐1* with age correlate well with our findings of reduced meiotic activity as male worms age. Meiotic activity is inhibited during STS stress, so to establish if STS stress also affects the transcriptional activities of APC/C subunit genes, we analyzed their transcript levels upon STS treatment. We found that, except for *emb‐30* and *fzy‐1*, STS stress did not reduce transcript levels for other subunit genes we examined (Figure 5b). In addition, we analyzed ribosomal translation under STS stress by isolating polysomes from the starved male worms and comparing their polysomal RNA profiles. We found that, with few exceptions, polysomal transcript levels for most APC/C genes were reduced by 10%–30% upon STS treatment (Figure 5b). However, the *fzy‐1* transcript levels in polysomes were markedly reduced (by more than 60%) upon STS treatment. These results suggest that aging and STS stress together may reduce expression of *fzy‐1*, which may in turn decrease APC/C activity.

To further examine if STS stress exerts a hormetic effect on the expression of *fzy‐1* in aged *C. elegans*, we isolated the testes from aged STS‐stressed male worms and analyzed their mRNA levels of APC/C subunits by qRT‐PCR. We found that prior STS stress does indeed significantly enhance the expression of *fzy‐1* in the testes of aged males (Figure 5c). The mRNA levels of another APC/C coactivator, *fzr‐1/cdh‐1*, were also significantly enhanced, while the mRNA levels of most APC/C subunits were not enhanced (Figure 5c; Supporting Information Figure S5).

### 2.7 Overexpression of *fzy‐1*in testes increases degradation of IZY‐1‐GFP fusion protein and inhibits the age‐associated rapid decline of meiotic activity and spermatogenesis in adult male *C. elegans*

**Figure 6:**
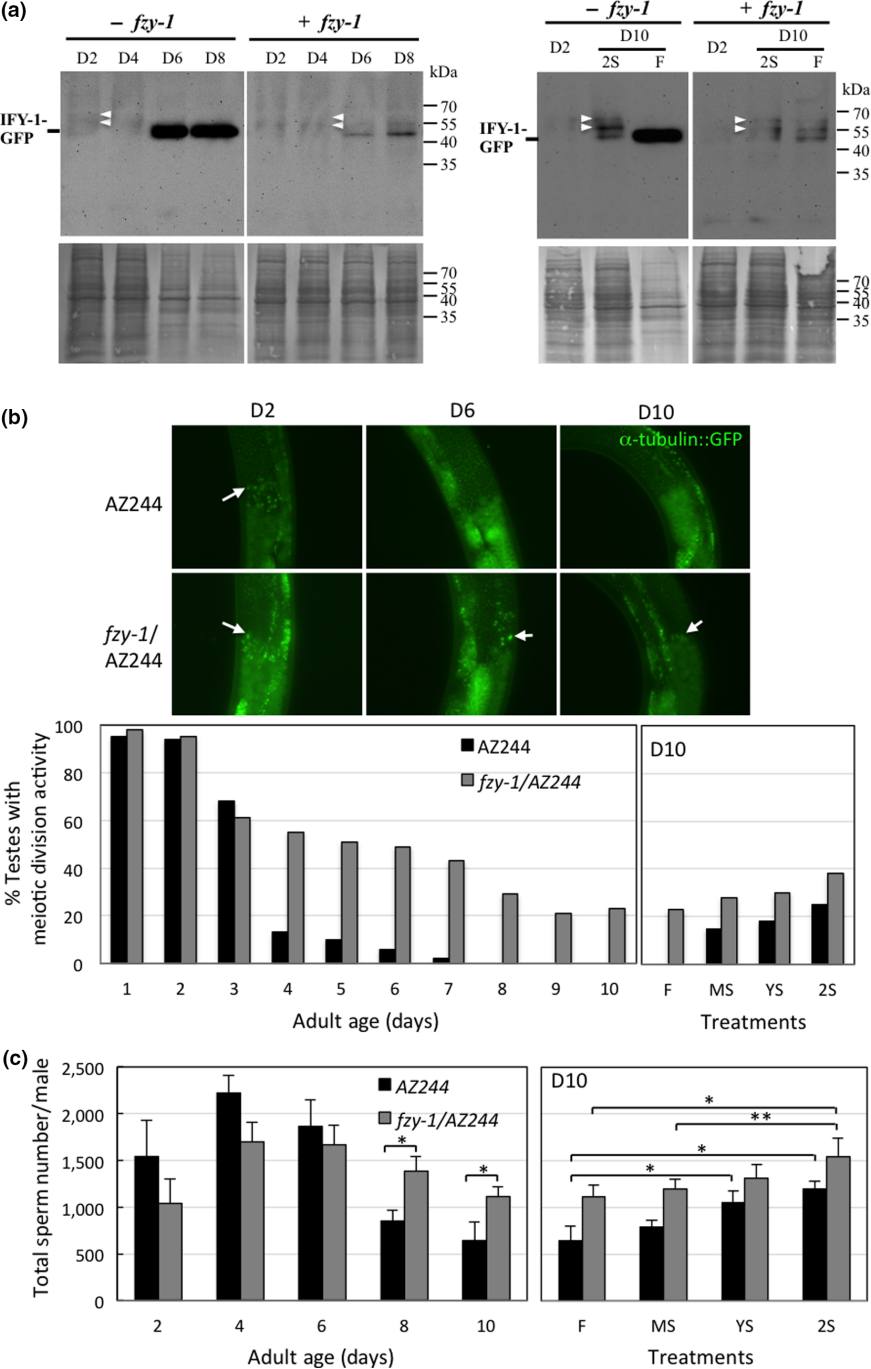
Transgenic expression of *fzy‐1*in gonads retards age‐associated decline in meiosis and spermatogenesis in adult male *C. elegans*. (a) Upper panel, western blotting analysis of IFY‐GFP fusion protein degradation in adult males of *fzy‐1*transgenic worms at each age and after repeated STS stress treatment (2S) as described in Figure 1a. White arrowheads indicate the super‐shifted bands of GFP signal. Lower panel, Coomassie blue staining of the same blots. (b) Meiotic activities in testes of *α‐tubulin/fzy‐1*transgenic adult male worms of different ages at 22°C, and of different STS stress‐treated as shown in Figure 1a. More than 50 worms from each age‐group or treatment group were live‐imaged. Upper panel, fluorescent live images of the division zone of adult male testes. Arrows indicate examples of α‐tubulin spindles. Lower panel, comparison of the percentage of meiotically active testes at each age and after STS stress treatment. (c) Sperm counts in transgenic adult male worms of different ages maintained at 22°C, and of different STS stress‐treated worms as shown in Figure 1a. Data represent mean ± *SD*, *n* = 10–12. Difference between two indicated groups: *, *p *≤ 0.01

Our results show that, from among APC/C subunits, coactivator *fzy‐1* is the only age‐regulated gene, and FZY‐1 is not only required to preserve sperm production under STS stress, but its expression levels are significantly increased by STS stress. We hypothesized that this STS‐induced hormetic effect on meiosis and spermatogenesis may be mediated by enhanced expression of FZY‐1 following stress exposure and that transgenic expression of *fzy‐1* to maintain its transcript levels during aging might protect spermatogenic activity from age‐related decline. Accordingly, we generated a transgenic *C. elegans* strain that stably expresses in gonads an FZY‐1‐GFP fusion protein driven by the *pie‐1* promoter (D'Agostino, Merritt, Chen, Seydoux, & Subramaniam, 2006). IFY‐1 is a securin, the protein ligand of FZY‐1, and is ubiquitinized by APC/C for degradation (Kitagawa, Law, Tang, & Rose, 2002). IFY‐1‐GFP fusion protein should appear as a single peptide in an SDS‐PAGE gel when a gonad lacks APC/C activity, whereas IFY‐1‐GFP fusion protein is likely ubiquitinized and degraded when a gonad possesses APC/C activity, so it might appear as multiple peptides of higher molecular weight before degradation. As shown in Figure 6a, IFY‐1‐GFP appeared as a single peptide in males at D6 and D8, whereas young males and D10 males stressed twice with STS presented markedly reduced levels and multiple peptides of higher molecular weight. This result supports that APC/C activity in testes declines rapidly with age, but can be attenuated by STS stress. We further introduced a *fzy‐1* transgene for stable expression in gonads to establish its effect on APC/C activity. As expected, transgenic expression of *fzy‐1* extended testis APC/C activity into later stages of adult life (Figure 6a). We then monitored the sperm‐producing capacity and meiotic division activity of male *fzy‐1* transgenic worms during aging. We observed that transgenic expression of *fzy‐1* markedly extended testis meiotic activity into later stages of adult life and retarded the age‐associated decline of sperm numbers (Figure 6b,c, left panels). Moreover, the hormetic effects of STS stress on increasing meiotic activity and sperm production could still be observed in aged *fzy‐1* transgenic males, though to a lesser extent than seen in control males (Figure 6b,c, right panels). Despite its beneficial effect on maintaining sperm production, transgenic expression of *fzy‐1* in gonads did not attenuate the age‐associated decline in copulatory activity (Supporting Information Figure S6).

## 3 DISCUSSION

Calorie restriction is an effective way to extend the lifespan of divergent species (L'opez‐LIuch & Navas, 2016). Chronic CR usually extends lifespan and prevents age‐associated fitness declines, whereas intermittent starvation regimes have generated inconclusive or even contradictory results (Anson, Jones, & de Cabod, 2005; Inness & Metcalfe, 2008; Xie et al., 2017). Interestingly, our study clearly demonstrates that one short‐term bout of starvation stress can elicit a hormetic effect to prevent age‐associated decline in spermatogenic activity of male *C. elegans*. Moreover, this STS stress‐induced hormetic effect is cumulative and lasting, supported by the evidence that adult males stressed once at 2–4 days of adult age still exhibited significantly higher spermatogenesis and several other physiological activities at 10 days of adult age than age‐matched non‐stress‐treated males. Furthermore, repetitive stress (two bouts) further enhanced this hormetic effect.

Calorie restriction extends lifespan, but it likely reduces reproductive capability in a trade‐off to save energy for somatic fitness (Grandison, Piper, & Partridge, 2009; Maklakov & Immler, 2017). However, our study clearly demonstrates that the STS stress‐elicited hormetic effects on vitality at poststress ages include reproductive fitness, indicating that starvation‐induced hormesis does not have direct reproductive costs for physical fitness and that there is adequate energy to promote both physical and reproductive fitness at a later stage. It is therefore tempting to speculate that STS stress‐induced hormesis may elevate energy capacity sufficiently to promote all physiological functions during the poststress period.

Upon food deprivation, meiotic division in the germ cells of testes is abolished, which should immediately halt the production of mature spermatids, thereby presumably preserving energy for somatic tissues during the stress period. Interestingly, although mitosis is a highly energy‐demanding process (Salazar‐Roa & Malumbres, 2017), we found that mitotic activity in testes was not inhibited or reduced under starvation, so it is not responsible for starvation‐associated reductions in sperm production. Instead, we provide evidence (Figure 4b) that meiosis is primarily targeted and inhibited by energy shortage signals to halt sperm production during stress. This result raises the possibility that, in adult male *C. elegans*, regulation of germ cell proliferation is not coupled to body and/or cellular energy status as is typically considered (Angelo & Van Gilst, 2009; McLeod, Wang, Wong, & Jones, 2010). It remains unclear why, during starvation, adult male worms still allocate precious energy to maintain germ cell proliferation. This scenario is similar to our previous finding that, during the initial period of starvation, resources are still diverted to increasing ribosome biogensis and global protein biosynthesis (Tan et al., 2011). One possibility is that STS stress‐induced hormesis is involved in maintaining mitotic activity during the stress period to ensure immediate recovery of sperm production after the stress has ended.

Surprisingly, we observed that meiotic division in the testes of male worms is dramatically reduced to barely detectable levels in most males only 5 days into their adult stage, whereas reduced mitotic activity only becomes obvious at 7 days of adult age and thereafter declines gradually with age. This finding indicates that meiotic activity of germ cells in adult male *C. elegans* is more sensitive to aging than mitotic activity. Thus, aging first targets meiosis to stop sperm production. Whether aging also later targets germ cell mitosis or this reduction in mitosis is merely due to feedback effects of reduced meiotic activity awaits further study.

Based on our results, both aging‐ and energy stress‐associated reductions in spermatogenesis are primarily due to inhibition of meiotic division, that is, at the final step of sperm production, which would appear to be a highly efficient way to regulate sperm production. When the final step of spermatogenesis is blocked, sperm production can be immediately and effectively inhibited, whereas if other upstream steps are blocked, downstream germ cells could still progress through meiotic division to form sperm. Furthermore, halted sperm production can be immediately restarted by lifting the inhibition on meiotic division. Therefore, since we found that STS stress‐induced hormesis significantly preserved meiotic activity in testes of stressed aged males, it is conceivable that the STS stress‐induced hormetic effect on meiotic activity is a means of counteracting the adverse effects of both aging and stress on spermatogenesis.

Among the genes considered crucial by our criteria for STS‐induced sperm production, deficiency or knockdown of some genes (such as *cdk‐1* and *plk‐1*) resulted in equivalent numbers or even more sperm at D10 than in D2 or D4 males. Since there is no age‐related decline in their sperm production as in that of wild‐type *N2* males, STS‐enhanced sperm production may be attenuated in aged males of these mutant worms. Although these genes are considered to be required in STS stress‐preserved spermatogenesis based on our criteria, they may be in fact not involved in the mechanism underlying STS‐induced hormesis. Thus, further study is needed to understand their role in promoting reproductive aging and to clarify whether they play a role in STS‐induced sperm production.

The mechanism by which STS‐induced hormesis counteracts the aging effect on meiotic division requires the activity of APC/C. Our molecular analyses revealed that the preservation of APC/C activity after STS stress may be due in part to enhanced expression at poststress ages of APC/C coactivator, FZY‐1/CDC‐20. Interestingly, except for *fzy‐1*, transcriptional expression levels of all other APC/C subunit genes do not decline with age, indicating that transcriptional regulation of *fzy‐1* is a target of aging and that reduced levels of *fzy‐1* transcript may mediate the inhibitory effect of aging on meiotic activity. This possibility is supported by our transgenic analysis in which we overexpressed *fzy‐1* in gonads, which prevented rapid loss of meiotic activity with age. However, since the activities of both FZY‐1/CDC‐20 are also regulated by other mechanisms, such as phosphorylation (Pesin & Orr‐Weaver, 2008), we cannot rule out the possibility that STS stress‐induced hormesis also simultaneously enhances other pathways to increase FZY‐1/CDC‐20 activity for APC/C activation.

Lastly, transgenic expression of *fzy‐1* in gonads increases sperm production but cannot attenuate the age‐associated decline of copulatory activity and fertilization efficiency, indicating that stable expression of *fzy‐1* may be beneficial primarily in spermatogenesis. Apart from sperm quality and quantity, for a male to sire progeny, a successful mating to securely deliver sperms for fertilization is required (Chatterjee et al., 2013). During mating, a male needs to execute a series of stereotyped actions involving the copulatory structure, physical strength, and precise coordination of body movement (Barr & Garcia, 2006). Aging is the major cause for the decline of activities of these mating‐associated physical and behavioral factors late into adult life (Chatterjee et al., 2013; Guo, Navetta, Gualberto, & García, 2012). Conceivably, the STS stress‐enhanced copulation and fertilization efficiency in aged male worms must involve hormetic effects on many, if not all, of these mating‐associated factors. It has also been reported that transient starvation during early adulthood has the hormetic effect of suppressing *unc‐103* mutation‐induced muscle contractions that cause uncoordinated movement during mating and result in failed copulation (LeBoeuf, Guo, & García, 2011). Taken together, the hormetic effect of STS stress on preventing age‐associated decline of male reproduction appears to be complex and broad. Nevertheless, for the age‐associated decline of sperm production, our study reveals that it is initiated by the dramatic decrease in APC/C activity resulting in limited meiotic division in germ cells of young males and that it can be attenuated by the STS‐elicited hormesis through up‐regulation of *fyz‐1* expression to maintain APC/C activity.

## 4 METHODS AND MATERIALS

### 4.1 Worm strains and generation of transgenic worms

Worms were maintained on NGM agar plates seeded with a lawn of *Escherichia coli* strain OP50 using standard procedures (Brenner, 1974). Temperature‐sensitive (ts) mutants were maintained at 15°C, whereas non‐temperature‐sensitive mutants were kept at 22°C. The worm strains used in this study included *N2*, AZ244, and the strains listed in Table 1, all purchased from the Caenorhabditis Genetics Center (CGC) at the University of Minnesota. All‐male populations of each strain were maintained by mating within every generation. To obtain the synchronized adult male worms, 20–25 mated hermaphrodites were allowed to lay eggs on a 6‐cm agar plate with a lawn of OP50 for 6 hr. Eggs were allowed to hatch and grow until they reached the first day of adult stage at the temperature indicated. Adult males were then isolated from hermaphrodites by hand picking or using a worm sorter (COPAS, Union Biometrica), based on the differences in body size and granularity between males and hermaphrodites.

For germline expression of the transgenes *fzy‐1*and *ify‐1*, the coding sequences of each gene were tagged with GFP at the C’ termini before being subcloned into the vector pID2.02 (D'Agostino et al., 2006). The transgenic worms with genome‐integrated transgenes were generated by a DNA microinjection and X‐ray irradiation approach.

### 4.2 Short‐term starvation (STS) stress treatment

The STS treatments we applied are depicted in Figure 1a. Synchronized male worms were collected on their first day (D1) of adulthood and transferred into phosphate‐buffered saline (PBS) supplied with OP50 bacterial food in a 12‐well plate. These worms were then starved on the following day (D2). Fed control worms (F) received OP50 bacterial food on a daily basis. Early‐ (YS) and mid‐stage (MS) STS stress‐treated groups received a 2‐day starvation treatment, either from D2 to D4 or D6 to D8, respectively. Worms subjected to repetitive stress (the 2S group) received both of these 2‐day starvation treatments. Worms of all groups were washed and supplied daily with fresh PBS and OP50, when applicable, and were collected at D10 for further analyses. For temperature‐sensitive mutant worms, adult males were first subjected to 25°C for 1 day at D2 to induce phenotypes before being subjected to STS stress treatment on D3 and then following the scheme shown in Figure 1a.

### 4.3 Survival rate monitoring

Two hundred D1 adult male worms were placed in a well of a 12‐well plate with 0.8 ml PBS and 15 µl freshly prepared OP50 bacteria (tenfold concentrate from overnight culture) at 15 or 22°C. Fresh PBS and OP50 bacteria were provided on a daily basis. Worms were scored as dead if they failed to move when poked. Dead worms were recorded and removed daily. Starvation treatments were started from D2 as described above for monitoring at 22°C. For survival monitoring at 15°C, STS treatment was extended to 3 days and also involved 3‐day recovery if worms were subjected to repetitive STS stress.

### 4.4 Body thrashing activity assay

*Caenorhabditis elegans* thrash their bodies from side to side in liquid environments without forward or backward motion. To monitor thrashing activity, five adult male worms were placed in a well of a 12‐well plate filled with fresh PBS and video‐recorded for 10 min at 22°C. A total of 30 worms were video‐recorded for each group. Numbers of full body swings (left to right and back again) were counted for individual worms.

### 4.5 Sperm production assay

Intact testes (Figure 3a) from male worms of different ages and STS treatments were dissected out in PBS and fixed in 0.5% paraformaldehyde at 4°C overnight. The intact testes were then transferred to a new microscope slide and stained with Hoechst 33342 (1 μg/ml) to visualize nuclei. Images of sperm were taken under a ZEISS Imager A1 microscope. Total numbers of sperm per testis were counted manually from the captured images. Ten to 12 intact testes were used for sperm counts for each group.

### 4.6 In vitro sperm activation

Live male worms were directly dissected in sperm activation medium (pH 7.7; 60 mM triethanolamine, 50 mM HEPES, 50 mM NaCl, 25 mM KCl, 5 mM CaCl_2_, 1 mM MgSO_4_, and 10 mM glucose). A pseudopod would form if sperm were activated. We counted sperm cells with and without pseudopods to calculate the percentage of sperm activation.

### 4.7 RNA‐seq and qRT‐PCR

For RNA‐seq, worms of the indicated ages were collected and total RNA was extracted using a TRIzol reagent (Invitrogen Life Technology). The mRNAs were sequenced in an Illumina HiSeq 2000 sequencer. For qRT‐PCR, a single worm approach was used for the isolated testes. Briefly, 40 testes were dissected out directly into 5 µl lysis buffer (5 mM Tris pH 8.0, 0.5% Triton X‐100, 0.5 Tween 20, 0.25 mM EDTA, 1 mg/ml proteinase K) before adding 10 µl of 40 U RNase inhibitor and 3 U DNase I mixture. The lysate was incubated at 37°C for 10 min and then at 75°C for 10 min to inactivate DNase I. To generate cDNA, the whole lysate was reverse‐transcribed with oligo dT primer (50 µM) in a final volume of 25 µl at 25°C for 10 min, 37°C for 2 hr, and then 85°C for 5 min.

qPCR was performed using SYBR green on an ABI 7500 Fast Real‐time PCR System (Applied Biosystem). We used 0.5 µl of cDNA solution for qPCR with each primer pair as indicated in Supporting Information Table S3. Each primer pair was checked for its melting curve before being used for qPCR. Relative quantification was computed as the threshold number of cycles of a target gene relative to that of the endogenous control *sgo‐1*gene.

### 4.8 EdU‐labeling assay for mitotic activity

Live male worms were incubated in PBS with 10 μM EdU (Invitrogen) and bacterial food for the indicated period. Testes were dissected out, fixed and mounted onto slides and then incubated in freshly prepared Click‐IT reaction cocktails (Invitrogen) for 30 min in the dark at room temperature. After incubation, testes were washed in PBS for 5 min and then stained in Hoechst 33342 (1 μg/ml) to visualize nuclei.

### 4.9 Immunostaining of α‐tubulin for meiotic division activity

Testes were dissected out, fixed and mounted onto slides and then incubated with mouse anti‐α‐tubulin FITC‐conjugated antibody (DMA1, Sigma‐Aldrich) in the dark at room temperature. After incubation, testes were washed in PBS for 5 min, and Hoechst 33342 was added at a final concentration of 1 μg/ml to visualize nuclei. More than 50 testes from each age‐group were analyzed for the presence of α‐tubulin spindles. Testes with at least one α‐tubulin spindle in their division zone are considered meiotically active.

### 4.10 RNAi knockdown

RNA interference was conducted by feeding worms with HT115 (DE3) bacteria (Fire Lab) carrying a recombinant L4440 vector. The RNAi bacterial clones, each carrying an open reading frame of *apc‐2*, *apc‐10*, *apc‐11,* or *mat‐4*, were purchased from the *C. elegans* RNAi Feeding Library (Openbiosystem). RNAi clones carrying the *gfi‐3* or *fzr‐1* ORF were constructed by integrating the respective partial cDNA sequences into L4440 vector. HT115 bacteria carrying the L4440 vector only and OP50 bacteria were used as nontargeting controls. To perform RNAi knockdown, male worms were collected at D1, placed onto RNAi bacterial plates, and kept at 15°C for 3 days. Worms were then collected and washed several times with PBS before being transferred into PBS in a 12‐well plate to receive a starvation treatment on the following day (D4) at 15°C. After STS stress treatment, worms were continuously fed with the same RNAi bacteria (prepared freshly on a daily basis) at 15°C.

### 4.11 Western blotting

Worms were collected, washed several times with PBS, and stored at −80°C until analysis. Frozen worms were homogenized with RIPA buffer containing protease inhibitor cocktail. Protein concentrations were determined using DC protein assay (Bio‐Rad). Protein (20–50 µg) from each sample was resolved in a 12% SDS‐PAGE gel, transferred to a PVDF membrane (Millipore, IPVH00010), blotted with the antibody against GFP (Abcam) in 5% nonfat milk at 4°C overnight, and detected with an ECL system (Amersham). Coomassie blue staining of the blot was used to assess loading of protein samples.

### 4.12 ATP/ADP measurement

To prepare whole‐body lysates, we placed 200 worms in 100 µl PBS before subjecting them to one freeze‐thaw cycle, then boiling for 30 min, and sonication for 30 s. ATP levels were determined for whole‐body lysates using an ADP/ATP Bioluminescence Assay kit (ApoSENSOR, BioVision). We used a BCA protein assay (BioVision) to determine protein levels in whole‐body lysates.

### 4.13 Copulatory activity and fertilization efficiency analysis

Copulatory activity and fertilization ability in D10 STS stress‐treated male worms were evaluated by mating stress‐treated D10 males to spermatogenesis‐defective mutant hermaphrodites of the BA785 [*spe‐8(hc40)*] line and dumpy hermaphrodites (AV125 [*spe‐8(hc40)*; *dpy‐4(e1166)*]), respectively. Twenty mutant hermaphrodites were crossed 1:1 with stress‐treated D10 *N2* male worms for 48 hr on ≤0.5 cm diameter lawns of PP50 bacteria at 22°C. Mutant hermaphrodites were then individually placed in a well of a 12‐well NGM agar plate seeded with OP50. They were allowed to lay eggs at 22°C until dead, and their progeny were counted. To assess fertilization ability, outcrossed nondumpy progeny sired by *N2* males were counted.

### 4.14 Statistical analysis

Results are presented as mean ± standard deviation (*SD*). Two‐tailed Student's *t* tests were used for statistical analysis. *p* < 0.05 was considered statistically significant.

## PUBLICATION DISCLAIMERS

The findings and conclusions in this report are those of the author(s) and do not necessarily represent the official position of the Institute of Molecular Biology, Academia Sinica.

## CONFLICT OF INTEREST

None declared.

## AUTHOR CONTRIBUTIONS

Y.‐H.L. designed the study, interpreted the data, and wrote the manuscript; W.‐Y.C. and Y.‐C.L. performed the experiments. All authors approved the final version of the manuscript.
