# Supplementary material for: Short‐term starvation stress at young adult stages enhances meiotic activity of germ cells to maintain spermatogenesis in aged male Caenorhabditis elegans
Source: Aging Cell. 2019 Feb 28;18(3):e12930. doi: 10.1111/acel.12930 (PMC6516166; doi:10.1111/acel.12930)
Supplement: Supplementary file 1 [file ACEL-18-e12930-s001.doc]

**Table S1. References for the genes described in Table 1.**

| **Genes** | **References** |
| --- | --- |
| *gld-1* | Kimble, J. & Crittenden, S.L. (2005). Germline proliferation and its control. *WormBook*, ed. The *C. elegans* Research Community, *WormBook*, doi/10.1895/wormbook.1.13.1 |
| *gld-2* | Kimble, J. & Crittenden, S.L. (2005). Germline proliferation and its control. *WormBook*, ed. The *C. elegans* Research Community, *WormBook*, doi/10.1895/wormbook.1.13.1 |
| *cdk-1* | Boxem, M., Srinivasan, D.G., & van den Heuvel, S. (1999). The *Caenorhabditis elegans* gene *ncc-1* encodes a *cdc2*-related kinase required for M phase in meiotic and mitotic cell divisions, but not for S phase. *Development* *126*, 2227–2239. |
| *plk-1* | Chase, D., Serafinas, C., Ashcroft, N., Kosinski, M., Longo, D., Ferris, D.K., & Golden, A. (2000). The polo-like kinase [PLK-1](http://www.wormbase.org/db/seq/protein?name=PLK-1;class=protein) is required for nuclear envelope breakdown and the completion of meiosis in *Caenorhabditis elegans*. *Genesis 26*, 26–41. |
| *zim-1* | Phillips, C.M., & Dernburg, A.F. (2006). A family of zinc-finger proteins is required for chromosome-specific pairing and synapsis during meiosis in *C. elegans*. *Dev. Cell 11*, 817–829. |
| *cep-1* | Schumacher, B., Hofmann, K., Boulton, S., & Gartner, A. (2001). The *C. elegans* homolog of the p53 tumor suppressor is required for DNA damage-induced apoptosis. Curr. Biol. *11*, 1722–1727. |
| *mat-1* | Shakes, D.C., Sadler, P.L., Schumacher, J.M., Abdolrasulnia, M., & Golden, A. (2003). Developmental defects observed in hypomorphic anaphase-promoting complex mutants are linked to cell cycle abnormalities. *Development* *130*, 1605–1620. |
| *mat-2* | Shakes, D.C., Sadler, P.L., Schumacher, J.M., Abdolrasulnia, M., & Golden, A. (2003). Developmental defects observed in hypomorphic anaphase-promoting complex mutants are linked to cell cycle abnormalities. *Development* *130*, 1605–1620. |
| *mat-3* | Shakes, D.C., Sadler, P.L., Schumacher, J.M., Abdolrasulnia, M., & Golden, A. (2003). Developmental defects observed in hypomorphic anaphase-promoting complex mutants are linked to cell cycle abnormalities. *Development 130*, 1605–1620. |
| *emb-1* | Golden, A., Sadler, P.L., Wallenfang, M.R., Schumacher, J.M., Hamill, D.R., Bates, G., Bowerman, B., Seydoux, G., & Shakes, D.C. (2000). Metaphase to anaphase (*mat*) transition-defective mutants in *Caenorhabditis elegans*. J. Cell Biol. *151*, 1469–1482. |
| *emb-27* | Shakes, D.C., Sadler, P.L., Schumacher, J.M., Abdolrasulnia, M., & Golden, A. (2003). Developmental defects observed in hypomorphic anaphase-promoting complex mutants are linked to cell cycle abnormalities. *Development* *130*, 1605–1620. |
| *emb-30* | Furuta, T., Tuck, S., Kirchner, J., Koch, B., Auty, R., Kitagawa, R., Rose, A.M., & Greenstein, D. (2000). EMB-30: An APC4 homologue required for metaphase-to-anaphase transitions during meiosis and mitosis in *Caenorhabditis elegans*. *Mol. Biol. Cell 11*, 1401–1419. |
| *fzr-1* | Fay, D. S., Keenan, S. & Han, M. (2002). fzr-1 and lin-35/Rb function redundantly to control cell proliferation in C. elegans as revealed by a nonbiased  synthetic screen. *Genes Dev.* **16**,503 -517. |
| *apc-10 & tag-31* | Yeong, F.M. (2004) Anaphase-Promoting Complex in Caenorbabditis elegans. *Mol Cell Biol. 24*, 2215-2225. |
| *brc-1* | Adamo, A., Montemauri, P., Silva, N., Ward, J.D., Boulton, S.J., & La Volpe, A. (2008). [BRC-1](http://www.wormbase.org/db/get?name=WP:CE43190;class=Protein) acts in the inter-sister pathway of meiotic double-strand break repair. *EMBO Rep*. *9*, 287-292. |
| *cul-2* | Feng, H., Zhong, W., Punkosdy, G., Gu, S., Zhou, L., Seabolt, E.K., & Kipreos, E.T. (1999). CUL-2 is required for the G1-to-S phase transition and mitotic chromosome condensation in *Caenorhabditis elegans*. *Nat. Cell Biol. 1*, 486–492. |
| *mei-1* | Yang, H., McNally, K., & McNally, F.J. (2003). [MEI-1](http://www.wormbase.org/db/get?name=WP:CE32479;class=Protein)/katanin is required for translocation of the meiosis I spindle to the oocyte cortex in *C elegans*. Dev. Biol. *260*, 245–59. |
| *cdc-25.3* | [Sung, M.](https://www.wormbase.org/resources/person/WBPerson37908), [Kawasaki. I.](https://www.wormbase.org/resources/person/WBPerson1148), & Shim, Y. (2017). Depletion of cdc-25.3, a C. elegans ortholog of cdc25, increases physiological germline apoptosis.*FEBS Lett*. 591, 2131-2146. |
| *ceh-18* | Rose, K.L., Winfrey, V.P., Hoffman, L.H., Hall, D.H., Furuta, T., & Greenstein, D. (1997). The POU gene *[ceh-18](http://www.wormbase.org/db/gene/gene?name=ceh-18)* promotes gonadal sheath cell differentiation and function required for meiotic maturation and ovulation in *Caenorhabditis elegans*. *Dev. Biol*. *192*, 59–77. |
| *spe-26* | [Varkey, J. P.](https://www.wormbase.org/resources/person/WBPerson669), [Muhlrad, P. J.](https://www.wormbase.org/resources/person/WBPerson438), [Minniti, A. N.](https://www.wormbase.org/resources/person/WBPerson1279), [Do, B-K.](https://www.wormbase.org/resources/person/Do%20B-K), & [Ward, S.](https://www.wormbase.org/resources/person/WBPerson680) (1995). The Caenorhabditis elegans spe-26 gene is necessary to form spermatids and encodes a protein similar to the actin-associated proteins kelch and scruin. *Genes Dev, 9*, 1074-86. |

**Table S2. Orthologs of APC/C subunits and its co-activators in mammals and *C. elegans***

| **Mammals** | ^a^ ***C. elegans* orthologs** | **Required for STS stress-preserved spermatogenesis** |
| --- | --- | --- |
| APC1 | MAT-2 | Yes |
| APC2 | APC-2 | No |
| APC3 | MAT-1 | Yes |
| APC4 | EMB-30 | Yes |
| APC5 | GFI-3; SUCH-1 | No |
| APC6 | EMB-27 | Yes |
| APC7 | ^b^ NI | ⎯ |
| APC8 | MAT-3 | Yes |
| ^c^ NI | NI | ⎯ |
| APC10 | APC-10 | No |
| APC11 | APC-11 | No |
| APC12 | MAT-4 | Yes |
| APC13 | NI | ⎯ |
| ^d^ NI | NI | ⎯ |
| APC15 | NI | ⎯ |
| APC16 | EMB-1 | Yes |
| NI | APC-17 | Yes |
| **Co-activators** | | |
| CDC20 | FZY-1 | Yes |
| CDH1 | FZR-1 | Yes |

^a^ References:

Shaye, D.D., & Greenwald, I. (2011). OrthoList: a compendium of C. elegans genes with human orthologs. *PLoS One 6*, e20085.

Yeong, F.M. (2004) Anaphase-Promoting Complex in Caenorbabditis elegans. *Mol Cell Biol. 24*, 2215-2225.

WormBase. <https://www.wormbase.org/#012-34->6

^b^ NI, not identified.

^c,d^ Apc9 and Apc14 were identified in yeasts and not yet identified in mammals.

**Table S3. Oligonucleotide sequences used in qPCR analyses of mRNA levels of targeted genes.**

| **Genes** | **Primer sequences** | |
| --- | --- | --- |
| *sgo-1* | Forward | 5’- GCGTCGGAAAGGGTCGAAG |
|  | Reverse | 5’- GAGGTTTCCTAGCCGTTGG |
| *mat-2* | Forward | 5’- TGAGAAATTTGTTGGCGCGA |
|  | Reverse | 5’- CTTTGCCCAACCATCGTTCA |
| *apc-2* | Forward | 5’- GCGACCTGACACTGTACAAC |
|  | Reverse | 5’- GGCATCCAGTTTTCCCATCC |
| *mat-1* | Forward | 5’- TGACGATGGAGTGATACGGA |
|  | Reverse | 5’- ATGGGCGAGATCATGACCTC |
| *emb-30* | Forward | 5’- TTTGGGATACTGCGCTGAGA |
|  | Reverse | 5’- GTTCAAGAAATGCCTCGCCA |
| *emb-27* | Forward | 5’- CCGACATTGGCTGATGAAGG |
|  | Reverse | 5’- GGTTCTGCCGATTTCAAGCA |
| *mat-3* | Forward | 5’- TGGGAAGCCTTGTCTCGATT |
|  | Reverse | 5’- GTCACCAGTTGTTCGGCTTT |
| *apc-10* | Forward | 5’- CCACTCCGAGTCATGTCACT |
|  | Reverse | 5’- GTGCTTCCAACACTGCTGAG |
| *apc-11* | Forward | 5’- TGTCGATGAGCAGTTCTGAAC |
|  | Reverse | 5’- GCGACAAATTCCCAGAACCA |
| *emb-1* | Forward | 5’- TGTACCCATTCCACGTAGCA |
|  | Reverse | 5’- GCAAGTGACGCAGAAGATCT |
| *apc-17* | Forward | 5’- CAAGACAAGCGGCTACCATC |
|  | Reverse | 5’- TCTTGTGTCTCTCGTGCCAT |
| *fzy-1* | Forward | 5’- CTTGCTACCCAACGCAACTT |
|  | Reverse | 5’- CGGTAAGAGCTCCAGTGGAT |
| *fzr-1* | Forward | 5’- GGAGGCCAAGCAACAACTAC |
|  | Reverse | 5’- ACGATACTGATGCCCAACCA |
| *msp-3* | Forward | 5’- GACTTGGAGTTGATCCACCATG |
|  | Reverse | 5’- GTTCTTACGACGAACCATACCG |
| *try-5* | Forward | 5’-CTACTGAAACTTTGGCAACCTG |
|  | Reverse | 5’-CAGATCCATATGAGACAATTGC |

**
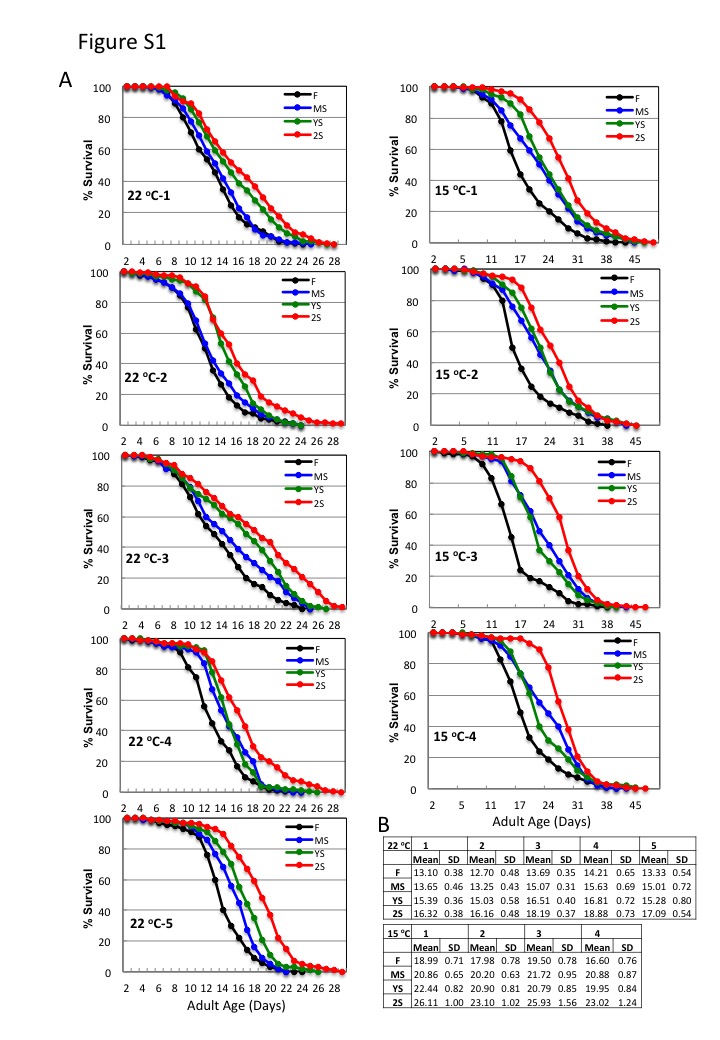
**

**Figure S1** Survival curves of all replicates for Figure 1b. (A) Survival curves of all replicates for *N2* adult male worms maintained at either 15 °C or 22 °C and that received various STS treatments as shown in Figure 1a. (B) Mean lifespan (days) under each STS treatment for each replicate shown in (A).


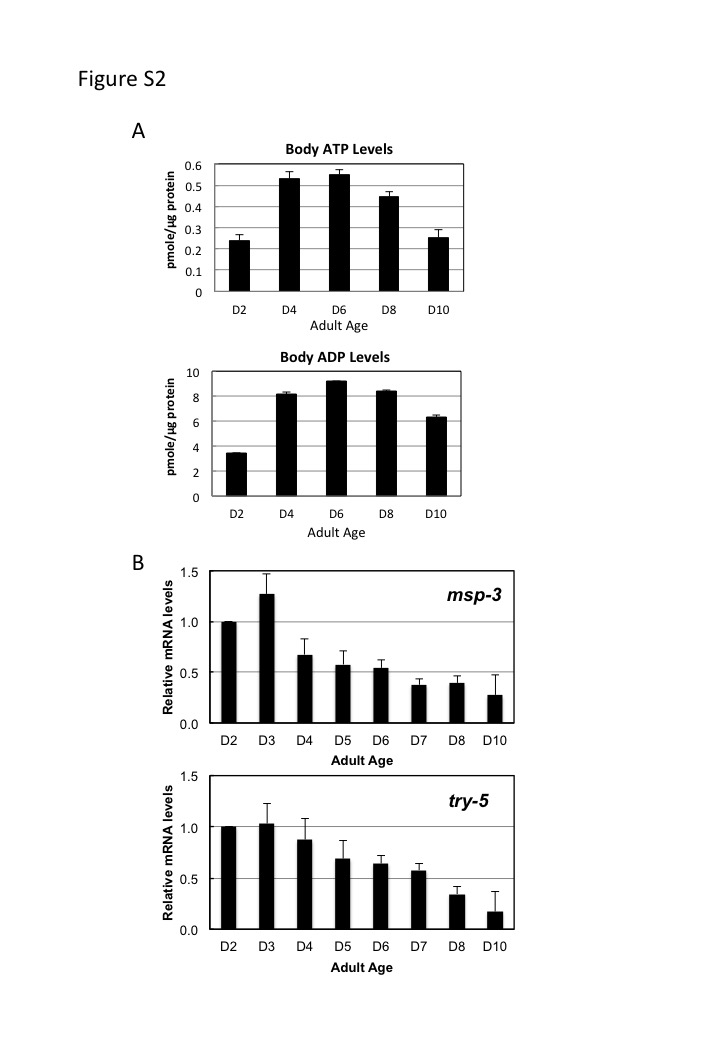


**Figure S2** (A) Body ATP levels decrease with age. Measurement of body ATP levels in adult male worms at different ages. Data represent mean ± SD, n=3. (B) The mRNA level of *msp-3* decreases with age. qRT-PCR analysis of *msp-3* mRNA levels in adult male worms at different ages. Data represent mean ± SD, n=4.


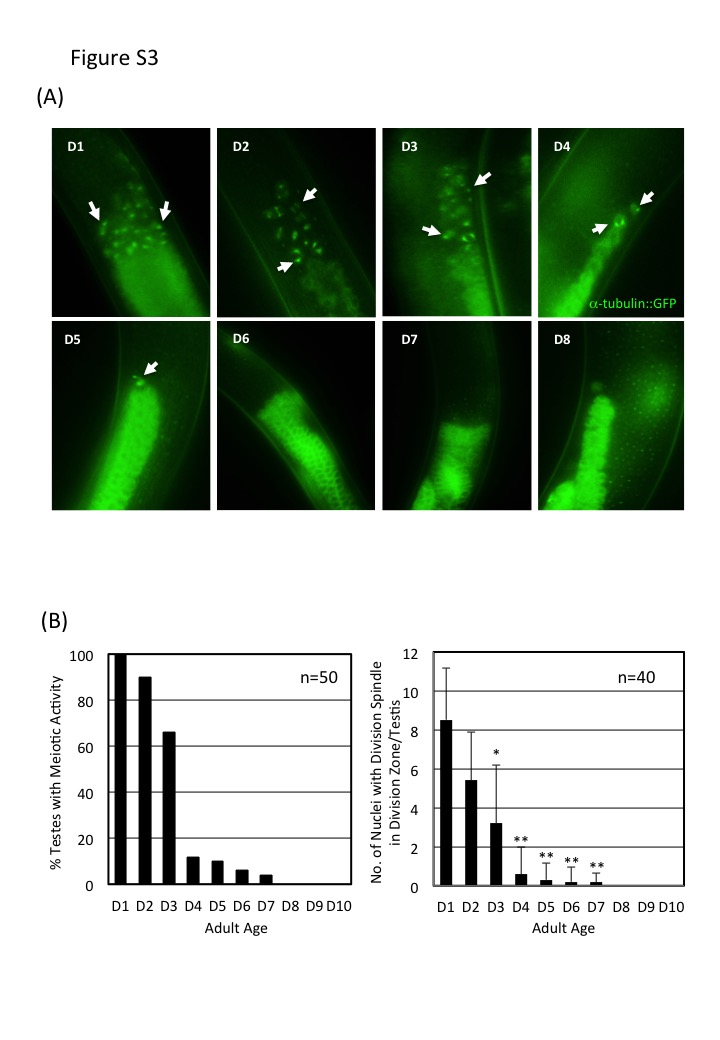


**Figure S3** Meiotic activity declines rapidly with age. (A) Fluorescent images of α-tubulin-GFP spindles in testes of live *AZ244* adult male worms of different ages. (B) Meiotic activities in testes of *AZ244* adult male worms of different ages and maintained at 22 °C. Fifty adult males from each age group were examined for the presence of α-tubulin-GFP spindles in their testes (Left panel). Testes with at least one α-tubulin spindle in their division zone were considered meiotically active. Right panel, number of nuclei containing α-tubulin-GFP spindles per testis. Data represent mean ± SD, n=40. *Different from day 1 level, *p<0.05*; **Different from the day 2 level, *p<0.001*.


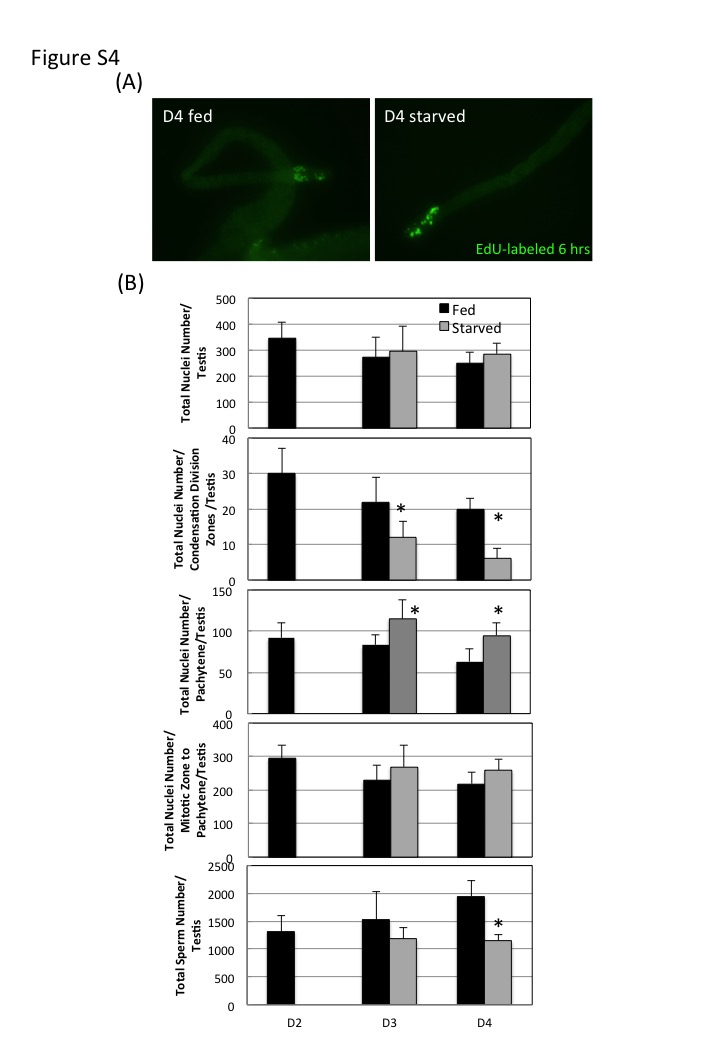


**Figure S4** Mitotic activity is not affected during STS stress treatment. (A) Fluorescent images of EdU-labeled nuclei in testes of D4 adult males that had been starved for 2 days and maintained at 22 °C. (B) Total sperm counts and numbers of nuclei in each zone of the testes isolated from D4 adult males that had been starved for 2 days and maintained at 22 °C. Data represent mean ± SD, n=10. *Different from fed control value of the same age cohort, *p<0.01*.


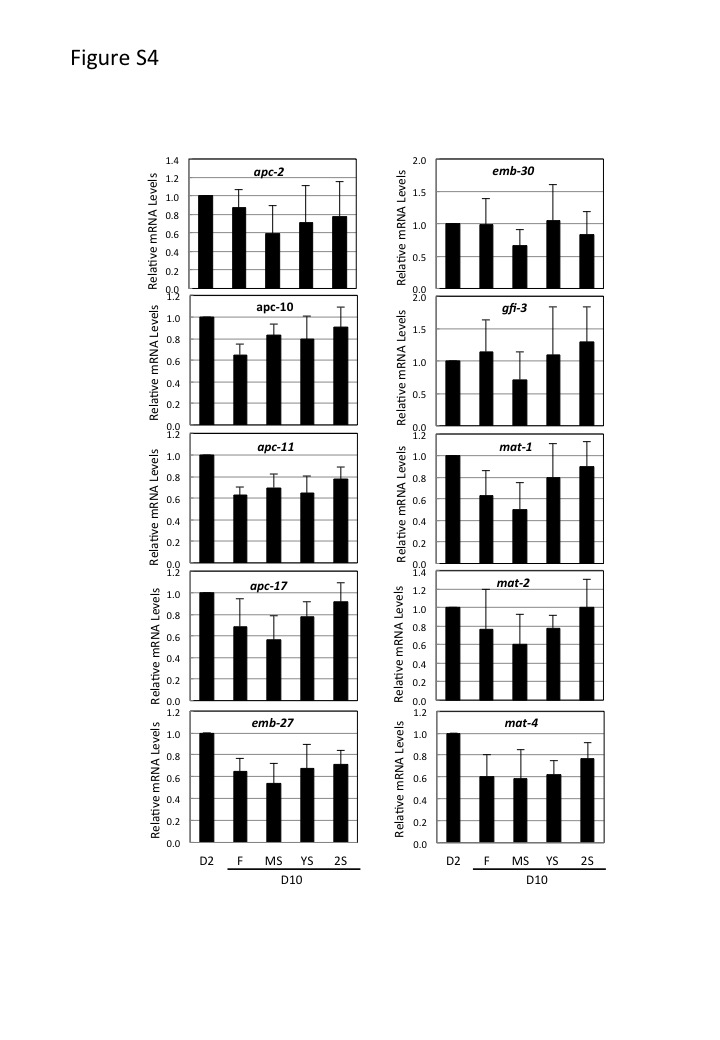


**Figure S5** qRT-PCR analysis of mRNA levels of APC/C subunits in testes isolated from D10 adult males that had been subjected to a previous STS stress treatment as shown in Figure 1a. Data represent mean ± SD, n=4.


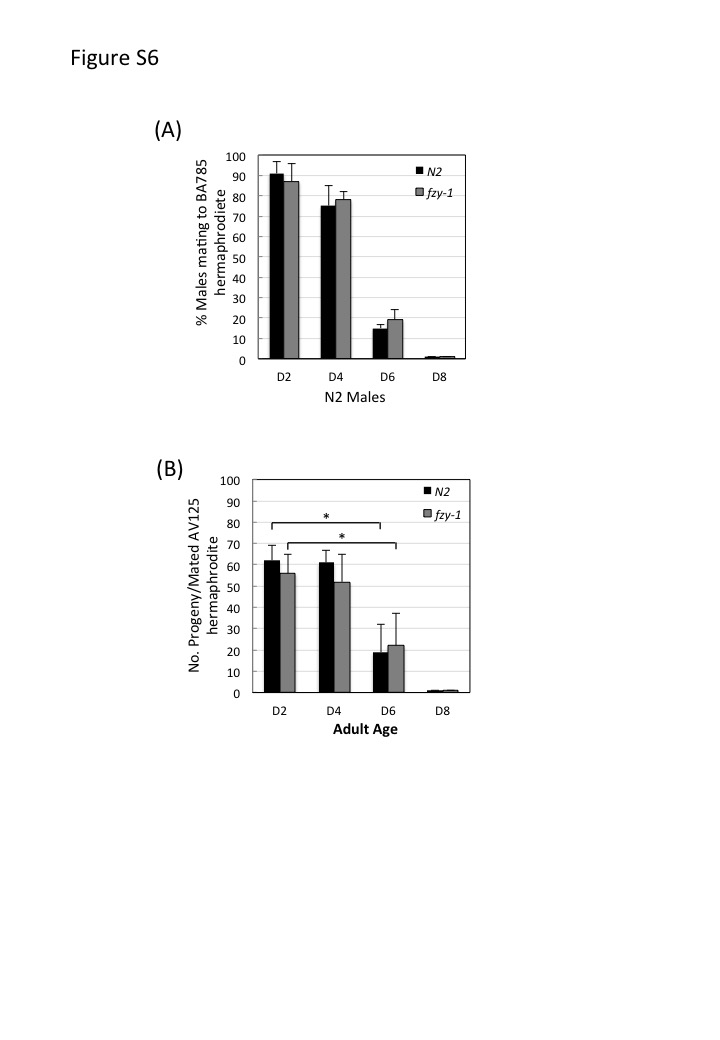


**Figure S6** (A) Copulatory activity in adult male worms of different ages. Data represent mean ± SD, n=3. (B) Fertilization ability in adult male worms of different ages. Data represent mean ± SD, n=10~12. Difference between two indicated groups: *, *p<0.001*.
